# Preparation and Characterization of Carbamazepine Cocrystal in Polymer Solution

**DOI:** 10.3390/pharmaceutics9040054

**Published:** 2017-12-01

**Authors:** Hao Zhang, Ying Zhu, Ning Qiao, Yang Chen, Linghuan Gao

**Affiliations:** 1 College of Materials Science and Engineering, North China University of Science and Technology, Tangshan 063210, China; zngzhang720@163.com (H.Z.); YingZhu20171003@163.com (Y.Z.); chenyang@ncst.edu.cn (Y.C.); 2 Hebei Provincial Key Lab of Inorganic Nonmetallic Materials, College of Materials Science and Engineering, North China University of Science and Technology, Tangshan 063210, China; 3 College of Basic Medical Sciences, North China University of Science and Technology, Tangshan 063210, China; gaolinghuan@ncst.edu.cn

**Keywords:** cocrystal preparation, polyvinyl pyrrolidone, carbamazepine, nicotinamide, saccharin

## Abstract

In this study, we attempted to prepare carbamazepine (CBZ) cocrystal through the solution method in ethanol-water solvent mixture (volume ratio 1:1) and polyvinyl pyrrolidone (PVP) solution. Nicotinamide (NIC) and saccharin (SAC) were selected as cocrystal coformers. Cocrystal screening products were characterized by Fourier Transform Infrared Spectroscopy (FTIR), Differential Scanning Calorimetry (DSC), and Powder X-ray Diffraction (PXRD) techniques. Characterization results show that in ethanol-water solvent mixture, pure CBZ-NIC cocrystal can be prepared, while CBZ-SAC cocrystal cannot be obtained. The addition of PVP can inhibit CBZ-NIC cocrystal formation and facilitate CBZ-SAC cocrystal formation.

## 1. Introduction

**Figure 1:**
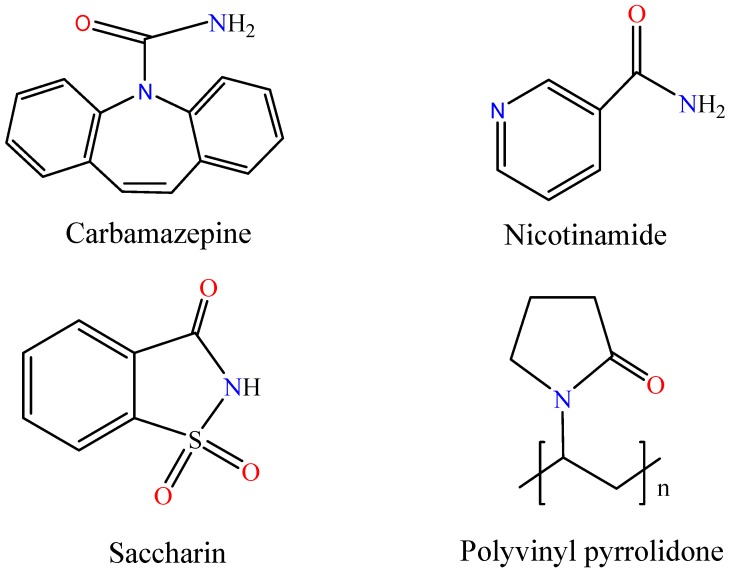
Molecular structure of carbamazepine, nicotinamide, saccharin, and polyvinyl pyrrolidone.

Pharmaceutical cocrystal is a crystalline material with two components present in definite stoichimetric amounts [1]. One of the pharmaceutical cocrystal components is an active pharmaceutical ingredient (API), and the other component is a cocrystal coformer (CCF). Pharmaceutical cocrystal is designed and prepared based on the supramolecular chemistry and crystal engineering theory. API and CCF are combined to form cocrystal through intermolecular interactions such as hydrogen bond (H-bond), π-π stacking force, van der Waals contact forces [2,3,4]. Recently, pharmaceutical cocrystal has been demonstrated to be a useful approach to modify physicochemical properties of drugs [5,6]. Among all the physicochemical properties, solubility and dissolution behavior are important properties which take great role in bioavailability of drugs [7]. A pharmaceutical cocrystal can be prepared with the intention to improve solubility behavior of an API without affecting its intrinsic molecular structure. For biopharmaceutics classification system class II (BCS II) drugs, which have high permeability and low solubility, pharmaceutical cocrystal has becoming a popular method to improve its solubility [8]. Carbamazepine (CBZ, IUPAC name 5H-dibenz (b,f) azepine-5-carboxamide, CBZ) is a typical BCS II drug whose bioavailability is limited by its relative low solubility. A high dose is usually administered to achieve the expected treatment effect. During the last decade, CBZ cocrystal has been studied to improve its solubility behavior [9,10,11,12,13,14]. Two main methods for cocrystal preparation are the grinding method and the solution method. It is difficult to obtain cocrystal product with high purity and good quality using the grinding method [15,16,17]. The solution method has been applied in many cases to prepare cocrystal [18,19]. In the solution method, the selection of solvent is very important. The ideal solvent for the solution cocrystallization process is one in which API and CCF has similar molar solubility. Poorly water soluble APIs, such as CBZ, API, and CCF, usually have unequivalent molar solubility in water. The cocrystallization through evaporation of an equimolar solution may result in crystallizing a single cocrystal component or a mixture of individual component and cocrystal. In order to solve this problem, the reaction cocrystallisation (RC) approach has been adopted. In the RC experiment process, the less soluble cocrystal component should be added into saturated or nearly saturated solutions of the other cocrystal component. When the solution becomes supersaturated with respect to cocrystal, the cocrystal product will gradually precipitate. Compared with the evaporation of an equimolar solution method, the experimental operation of RC method is relatively more complex [20,21]. Another method is cocrystallization with solvent mixture. In this method, a solvent mixture is used for the solution-mediated cocrystallization process. Solvent mixtures can reduce the solubility differences between cocrystal components; facilitate nucleation rate and provide improved transformation kinetics for cocrystallization [22,23,24]. Di Profio has successfully prepared carbamazepine-saccharin cocrystal in water/ethanol solvent mixtures by membrane-based crystallization technology [25]. In this study, ethanol-water solvent mixture was also applied to try to prepare CBZ cocrystal through a combination of solution evaporation and the cooling crystallization method. Meanwhile, a polymer additive was added in the solution to investigate the effect of polymer on pharmaceutical cocrystal formation. Previous research studied the effects of polymers on cocrystal stability and dissolution process. In some case, selected polymers can maintain structure stability of certain cocrystals and inhibit the precipitation of individual cocrystal components during the dissolution process [26,27,28,29]. From the point of view of dissolution equilibrium, cocrystal preparation via the solution method and cocrystal dissolution can be seen as the two directions of the dissolution equilibrium. Considering that polymers could impart structure stability to cocrystal during its dissolution in some cases, it is expected that the addition of polymer may help cocrystal formation through solution method. In this paper, the effects of polymer on cocrystal preparation process was studied. CBZ was selected as model drug, nicotinamide (NIC, IUPAC name pyridine-3-carboxamide) and saccharin (SAC, IUPAC name 1,1-dioxo-1,2-benzothiazol-3-one) as CCF, and polyvinyl pyrrolidone (PVP) as the representative polymer. The molecular structure of CBZ, NIC, SAC and PVP are shown in Figure 1. PVP is widely used in pharmacy industry as excipient [27,30,31,32,33,34]. In the macromolecule structure of PVP, it has both a hydrophilic and hydrophobic group. It is highly soluble in water and ethanol. During the cocrystallization process, PVP will not precipitate from the solution due to its high solubility and low amount of addition. It can stay in solution as its original dissolved state.

In this study, we attempted to prepare 1:1 molecular ratio cocrystal of CBZ-NIC and CBZ-SAC in 1:1 volume ratio of ethanol-water solvent mixture with and without PVP. Cocrystal screening products were characterized by Fourier Transform Infrared Spectroscopy (FTIR), Differential Scanning Calorimetry (DSC), and Powder X-ray Diffraction (PXRD). The effects of PVP on CBZ cocrystal formation process have been fully discussed.

## 2. Materials and Methods

### 2.1. Materials

CBZ (>99% purity) was purchased from Jianglai Reagent Co., Ltd. (Shanghai, China) and used as received. Nicotinamide (NIC, ≥99% purity) and saccharin (SAC, ≥98% purity) were obtained from Aladdin Industrial Co. (Shanghai, China) and used as received. Polyvinylpyrrolidone K30 (PVP) purchased from Tianjin Guangfu Fine Chemical Research Institute (Tianjin, China) was used as received. Ethanol from Yongda Chemical Reagent Co., Ltd. (Tianjin, China) was analytical grade and used as received. Potassium bromide (KBr, ≥99% purity) purchased from Jinke Chemical Reagent Co., Ltd. (Tianjin, China) was dried for 24 h at 50 °C and finely grounded just before use.

### 2.2. Sample Preparation 

#### 2.2.1. Preparation of CBZ-NIC, CBZ-SAC Cocrystal in Ethanol-Water Solvent Mixture

A mixture of CBZ (472.6 mg, 2 mmol) and NIC (244.3 mg, 2 mmol) (or SAC, 366.3 mg, 2 mmol) was added in 20 mL ethanol-water solvent mixture (with volume ratio of 1:1) in a 50 mL crystallizer flask. The flask was sealed and stirred under 50 °C for 20–50 min. After complete dissolution, the stirring and heating of the flask was stopped. The clear solution was cooled down slowly to room temperature. During the cooling process, a small amount of fine crystal was precipitated in the bottom of the flask. Then the solution was placed in a fume hood and allowed to evaporate slowly. After 24 h, about two fifths of the solvent has been evaporated, and more solid product precipitated. The solution was filtered and the solid residue was collected and dried for 12 h under vacuum. CBZ-NIC cocrystal and CBZ-SAC cocrystal screening product prepared in ethanol-water solvent mixture were obtained through the above procedures. The two cocrystal screening products were abbreviated as CBZ-NIC-E/W and CBZ-SAC-E/W, respectively.

#### 2.2.2. Preparation of CBZ-NIC, CBZ-SAC Cocrystal in PVP Solution

The PVP solution was prepared by dissolving 80 mg PVP into 20 mL ethanol-water solvent mixture. We tried to prepare the CBZ-NIC cocrystal and CBZ-SAC cocrystal in the PVP solution through the same procedures described in Section 2.2.1. The two cocrystal screening products were abbreviated as CBZ-NIC-PVP and CBZ-SAC-PVP respectively.

### 2.3. Methods

#### 2.3.1. Solubility Test

The solubility of CBZ, NIC, and SAC in all solvents used in this study (water, ethanol, and ethanol-water mixture with and without PVP) was tested at room temperature, respectively. Excess amount of solid samples were added into 10 mL solvent and vortexes for 20 s. The suspension was placed in horizontal air-shaking bath at 100 rpm for 24 h. Aliquots were filtered and diluted properly for determination of the concentrations of tested samples by Lambda 750S UV/Vis/NIR Spectrometer (PerkinElmer, Waltham, MA, USA). Concentration of CBZ, NIC, and SAC has been measured under the wavelength of 285, 260, and 276 nm respectively. The experiments were carried out in triplicate.

#### 2.3.2. Fourier Transform Infrared Spectroscopy (FTIR)

IR spectra of all CBZ cocrystal screening product were characterized by a Bruker VERTEX 70 FTIR spectrometer (Bruker Corporation, Karlsruhe, Germany). The spectrometer resolution is 2 cm^−1^; spectra recorded from 4000 to 400 cm^−1^ and number of runs per spectrum 5. The tested sample was processed by compressing the mixture of sample and KBr into a thin disk (sample concentration 2 mg in 20 mg KBr).

#### 2.3.3. Differential Scanning Calorimetry (DSC)

Thermal analyses of all CBZ cocrystal screening product were tested by a NETZSCH DSC 200F3 (NETZSCH Group, Selb, Germany). About 10 mg of tested samples were put in aluminum pans (NETZSCH, JYL0040 Φ 8 × 2.1 mm) and covered with a pierced lid. Measurements were carried out at a heating rate of 10 °C/min in the temperature range of 20 to 300 °C under a nitrogen flow rate of 20 mL/min.

#### 2.3.4. Powder X-ray Diffraction (PXRD)

PXRD analysis was performed by Rigaku D/max 2500 PC diffractometer (Rigaku Corporation, Tokyo, Japan). Instrument settings were: monochromatic Cu/Kα radiation (λ = 1.54180 Å), 40 kV/100 mA, 2θ region 3–40°, step of 0.02° and scan rate of 10°/min.

## 3. Results and Discussion

### 3.1. Solubility Test

The solubility of CBZ, NIC, and SAC in water, ethanol, and ethanol-water mixture with and without PVP was tested at room temperature. The concentration of samples in each solvent was measured by UV-VIS spectrometer and calculated based on corresponding calibration curve. Solubility data was shown in Table 1. CBZ is poorly soluble in water with a solubility of 0.00001 (mol fraction), while NIC has a relative high solubility in water. The solubility difference between NIC and CBZ has been significantly reduced from 0.09242:0.00001 in water to 0.01961:0.00011 in ethanol-water mixture. The solubility difference between SAC and CBZ has also been decreased in ethanol-water mixture. Solvent mixture and PVP have differential solubilization ability for different compounds. The addition of PVP can increase solubility of all compounds, and the solubility difference between NIC and CBZ has also been slightly increased compared with that in ethanol-water.

### 3.2. FTIR Analysis

**Figure 2:**
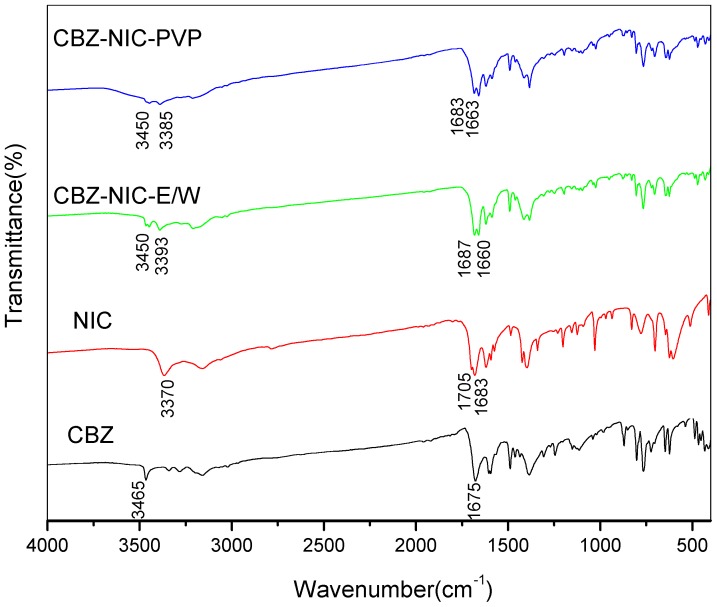
Infrared spectroscopy (IR) spectra of carbamazepine (CBZ), Nicotinamide (NIC), CBZ-NIC cocrystal screening product prepared in ethanol-water mixture (CBZ-NIC-E/W) and in polyvinyl pyrrolidone solution (CBZ-NIC-PVP).

The IR spectra for starting material of CBZ, NIC, and CBZ-NIC cocrystal screening products are shown in Figure 2. IR spectrum for CBZ has two characteristic peaks at 3465, 1675 cm^−1^, which correspond to the stretching of amine (NH_2_) and carbonyl (C=O) group of CBZ. The IR spectrum for NIC has a characteristic peak at 3370 cm^−1^ for the stretching of amine group and double characteristic peaks at 1705 and 1683 cm^−1^ for the stretching of carbonyl group. The IR spectrum of cocrystal screening product CBZ-NIC-E/W is different from that of the starting material of CBZ or NIC. The characteristic peak of CBZ in CBZ-NIC-E/W has red shift from 3465 and 1675 cm^−1^ to 3450 and 1660 cm^−1^, respectively. The characteristic peak of NIC in CBZ-NIC-E/W has a blue shift to 3393 and 1687 cm^−1^. The characteristic peak shift can be explained by the formation of H-bond, and the IR spectrum features of sample CBZ-NIC-E/W are in agreement with that of CBZ-NIC cocrystal [9,35]. IR characterization results can preliminarily demonstrate the formation of CBZ-NIC cocrystal in ethanol-water solvent mixture. For sample CBZ-NIC-PVP, its IR spectrum has shown some difference from that of CBZ and NIC. The CBZ characteristic peak has shifted to 3450, 1663 cm^−1^. However, an obvious peak at 1683 cm^−1^ corresponding for NIC can still be observed, which demonstrated the existence of NIC. IR results preliminarily demonstrate that the possibility of formation of pure CBZ-NIC cocrystal in PVP solutions has been eliminated.

**Figure 3:**
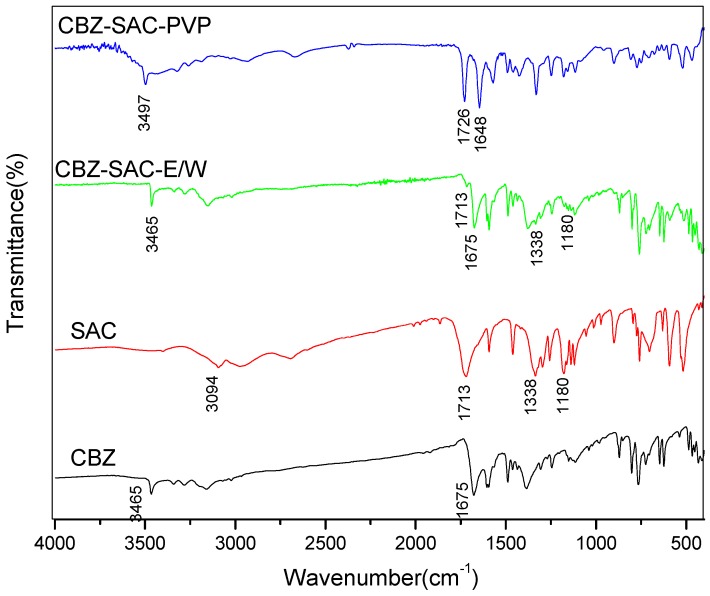
IR spectra of carbamazepine (CBZ), saccharin (SAC), CBZ-SAC cocrystal screening product prepared in ethanol-water mixture (CBZ-SAC-E/W) and in polyvinyl pyrrolidone solution (CBZ-SAC-PVP).

The IR spectra for CBZ, SAC, and CBZ-SAC cocrystal screening products are shown in Figure 3. SAC has characteristic peaks at 3094, 1713 cm^−1^ corresponding for stretch of amine and carbonyl group; peaks at 1338, 1180 cm^−1^ for the asymmetrical and symmetrical stretch of SO_2_ group. IR spectrum of sample CBZ-SAC-E/W has characteristic peaks for both CBZ and SAC, which demonstrate this sample is a physical mixture of CBZ and SAC. IR spectrum of sample CBZ-SAC-PVP is difference from that of single component of CBZ or SAC, which has shown characteristic peaks shift to 3497, 1648 cm^−1^ for CBZ, and peak at 1726 cm^−1^ for SAC. The features of sample CBZ-SAC-PVP is the same as that of CBZ-SAC cocrystal [11]. To conclude, CBZ-SAC cocrystal can be prepared in PVP solutions.

### 3.3. DSC Analysis

**Figure 4:**
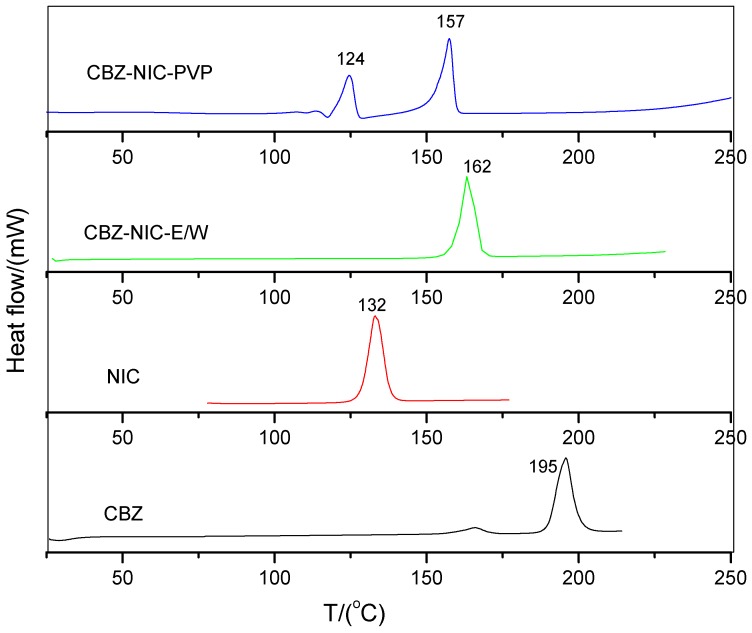
Differential Scanning Calorimetry (DSC) curves of CBZ, NIC, CBZ-NIC-E/W, and CBZ-NIC-PVP.

DSC curves of CBZ, NIC, CBZ-NIC-E/W, and CBZ-NIC-PVP were shown in Figure 4. As shown in Figure 4, DSC curve of starting material of CBZ and NIC have an endothermic peak attributed to melting around 195 °C and 132 °C, respectively. Sample CBZ-NIC-E/W has a single endothermal peak, which illustrates that this sample is a pure product with only one molecule structure. The single endothermal peak is around 162 °C, which is located between the melting point of NIC and CBZ. This peak should be assigned to the melting of CBZ-NIC cocrystal [9]. DSC thermal analysis further demonstrated the formation of pure CBZ-NIC cocrystal in an ethanol-water solvent mixture. Sample CBZ-NIC-PVP has two major thermal peaks around 124 and 157 °C in its DSC curve. The first thermal event around 124 °C could be due to the melting of NIC and followed by cocrystallization [14]. The second thermal event around 157 °C could be due to the melting of CBZ-NIC cocrystal, which is formed during the heating process [13]. Compared with pure NIC and cocrystal, the melting point of the two substances in the mixture both reduced. This could be explained by the existence of the second substance.

**Figure 5:**
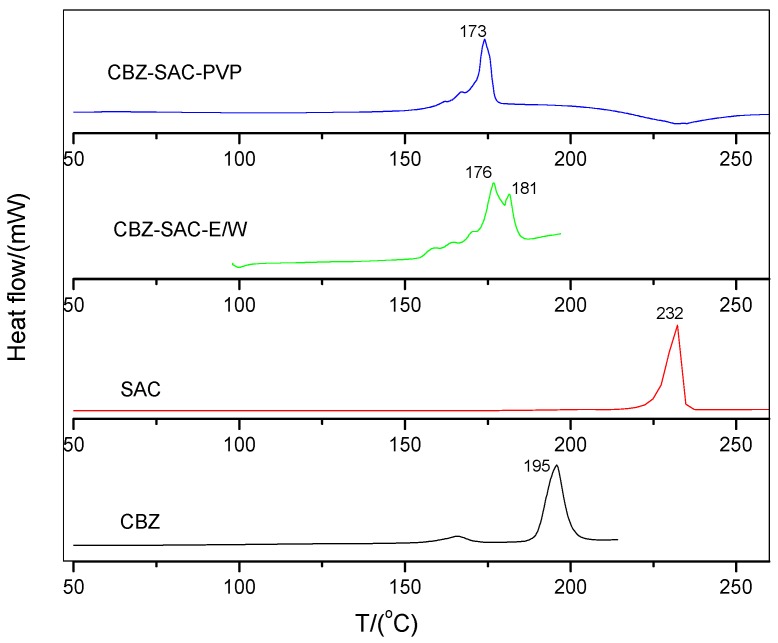
DSC curves of CBZ, SAC, CBZ-SAC-E/W, and CBZ-SAC-PVP.

DSC curves of CBZ, SAC, CBZ-SAC-E/W, and CBZ-SAC-PVP were shown in Figure 5. For sample CBZ-SAC-E/W, the two overlaid peaks of 176 and 181 °C indicate that this sample is a physical mixture. The two starting materials in the mixture melt one after another in a broad melting range at relative lower temperature compared to their pure state. Sample CBZ-SAC-PVP shows a single endothermal peak at 173 °C, corresponding to the melting point of CBZ-NIC cocrystal [36]. DSC analyses further demonstrate the formation of pure CBZ-SAC cocrystal in PVP solution.

### 3.4. PXRD Analysis

**Figure 6:**
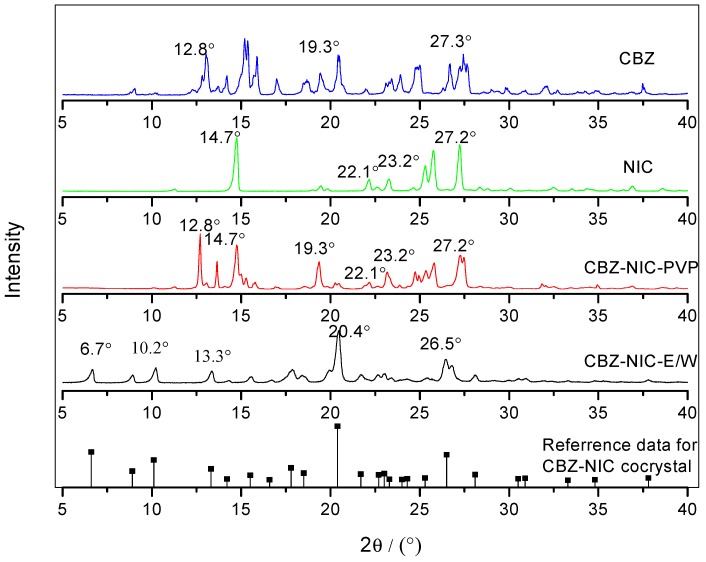
Powder X-ray diffraction (PXRD) spectra of CBZ, NIC, CBZ-NIC-PVP, CBZ-NIC-E/W, and CBZ-NIC-cocrystal.

The PXRD characterization results of CBZ-NIC cocrystal screening products and the original material of CBZ and NIC are shown in Figure 6. The reference data for CBZ-NIC cocrystal characteristic PXRD peaks are also shown in Figure 6. As shown in Figure 6, sample CBZ-NIC-PVP shows both CBZ and NIC features. Sample CBZ-NIC-E/W has new peaks at 2θ: 6.7°, 10.2°, and 13.3°, which are in good agreement with reported CBZ-NIC characteristic PXRD peaks [14,37]. Base on above characterizations, pure CBZ-NIC cocrystal was successfully prepared in 1:1 volume ratio ethanol-water solvent mixture, and there is no CBZ-NIC cocrystal formed in PVP solution, the product obtained in PVP solution is a mixture.

**Figure 7:**
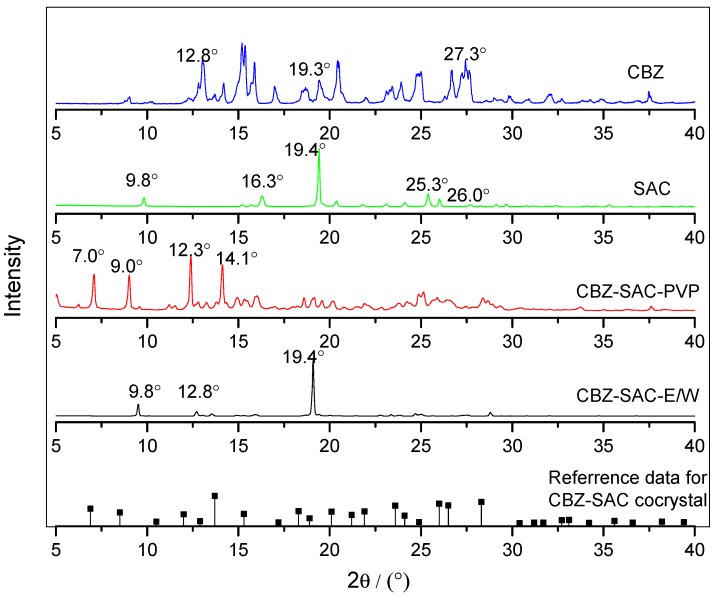
PXRD spectra of CBZ, SAC, CBZ-SAC-E/W, and CBZ-SAC-PVP.

The PXRD characterization results for CBZ-SAC samples are shown in Figure 7. Sample CBZ-SAC-E/W shows a mixture feature with both CBZ and SAC characteristic peaks. Sample CBZ-NIC-PVP has cocrystal features at 2θ: 7.0°, 9.0°, 12.3°, and 14.1° [12]. In sum, the CBZ-SAC cocrystal screening product prepared in ethanol-water is a mixture of CBZ and SAC, and the sample prepared in PVP solution is pure CBZ-SAC cocrystal.

### 3.5. Molecular Structures of CBZ Cocrystals

**Figure 8:**
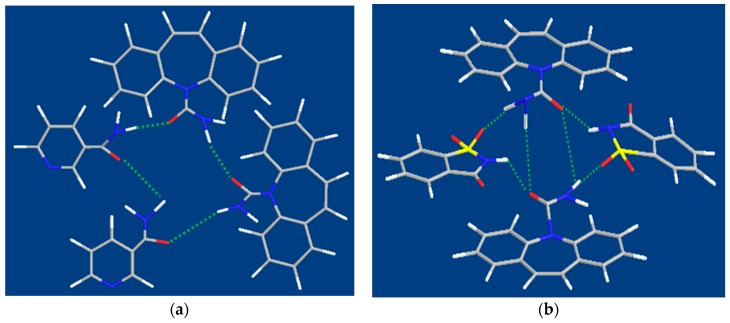
Illustration of the interaction between CBZ and coformer in cocrystal. (**a**) CBZ-NIC cocrystal (**b**) CBZ-SAC cocrsytal.

The above characterizations demonstrated the formation of CBZ-NIC cocrystal and CBZ-SAC cocrystal. The molecular structures of CBZ cocrystal are shown in Figure 8 [38,39]. CBZ-NIC cocrystal, Figure 8a, has an amide-to-amide structure. It is formed through N-H…O=C hydrogen bonds in which the carboxamide groups from both CBZ and NIC provide hydrogen bond donors and acceptors. For CBZ-SAC cocrystal, the coformer, SAC, has S=O group as hydrogen bond acceptor and N-H group as hydrogen bond donor. In CBZ-SAC cocrystal structure, Figure 8b, SAC is held by S=O…H-N and N-H…O=C hydrogen bonds with carboxamide groups of CBZ.

### 3.6. Discussion of the Effects of PVP on CBZ Cocrystal Preparation

**Figure 9:**
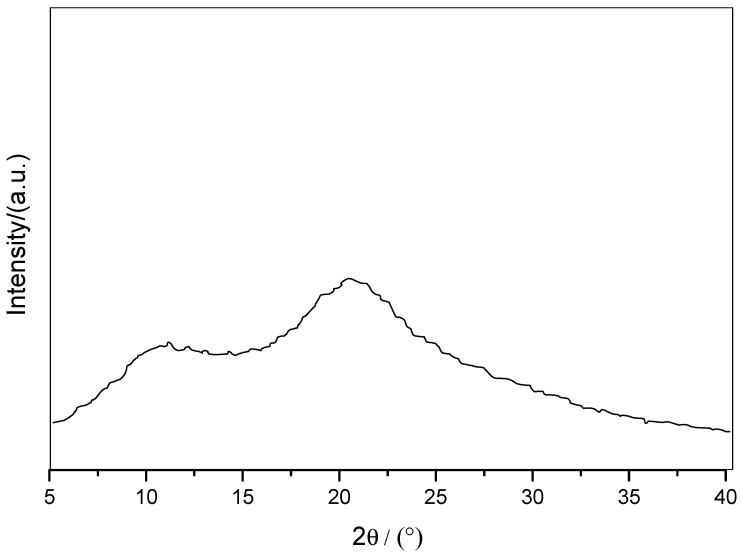
PXRD spectra of PVP.

In this study, the effects of PVP on CBZ-NIC and CBZ-SAC cocrystal formation process have been researched. PVP is one of the most researched polymers made from the monomer of *N*-Vinyl-2-pyrrolidone, which has been widely used in the food and pharmacy industries as an additive or excipient [27,30,31,32,33,34]. In the macromolecule structure of PVP, it has both flexible long carbon chain and side ring groups, pyrrolidinone, which offer some rigid property to PVP structure. The flexible chain and the rigid side group together can impart PVP adjustable steric structure properties. PVP is highly soluble in water, ethanol, and other polar solvents. The solubility of PVP in water/ethanol is about 1000 mg/mL. In this study, PVP could remain dissolved state in solution due to its high solubility. PVP has a broad endothermal peak around 50–100 °C [40,41,42], which is not observed in DSC curves of any cocrystal screening products. Besides, the PXRD spectrum of PVP, shown in Figure 9, has broad peaks around 11° and 20° corresponding to the PVP crystalline phase, although PVP has a poor crystallinity degree. While these characteristic broad peaks of PVP have not been found in PXRD spectrum of any samples prepared in PVP solutions. The test results of DSC and PXRD demonstrate that PVP did not precipitate during the cooling and slow evaporation cocrystal preparation process.

For CBZ-NIC cocrystal preparation, CBZ-NIC cocrystal screening product prepared in ethanol-water solvent mixture has been characterized as pure CBZ-NIC cocrystal. This demonstrates that a water-ethanol solvent mixture with 1:1 volume ratio is a promising solvent for CBZ-NIC cocrystal preparation. CBZ is poorly soluble in water, while NIC has a very high solubility in water. The solubility difference between the two cocrystal components can be decreased a lot in an ethanol-water solvent mixture, which facilitates the formation of CBZ-NIC complexation and the consequently precipitation of CBZ-NIC cocrystal. The sample prepared in PVP solution was characterized as a physical mixture, which indicated that the addition of PVP play an inhibition role in CBZ-NIC cocrystal formation. The solubility data show that PVP has no adverse effect on components dissolving. Both CBZ and NIC have some increase in their solubility in the PVP solution, but their solubility difference was also increased due to differential solubilizaiton of the two components. The inhibition on cocrystal formation could result from the interaction between the pyrrolidinone ring in PVP and the amide group in NIC. The carbonyl group in pyrrolidinone and the amine group in amide formed a relative strong connection, and this connection more competitive than that between CBZ and NIC. Thus, the complex and precipitation of CBZ-NIC cocrystal was affected by addition of PVP.

Although the solubility difference between CBZ and SAC has also been decreased in ethanol-water solvent mixture, the sample prepared in ethanol-water mixture was demonstrated as a physical mixture. In the PVP solution, the solubility difference has been further decreased slightly due to differential solubilization of two components. The sample prepared in the PVP solution was characterized as pure CBZ-SAC cocrystal. The addition of PVP facilitates the formation of CBZ-SAC cocrystal. The effect of PVP on CBZ-SAC cocrystal formation is opposite to that on the CBZ-NIC cocrystal. H-bond connection could also form between PVP and SAC, same as that formed between PVP and NIC. But the double ring structure in SAC molecule may generate larger steric hindrance. PVP may interfere the intermolecular interactions between CBZ and SAC but is not essential to disturb the formation of the CBZ-SAC cocrystal structure [43]. On the contrary, the addition of PVP induced the formation of CBZ-SAC cocrystal. It is assumed that PVP can help the nucleation of CBZ-SAC cocrystal.

## 4. Conclusions

In order to study the effect of PVP on cocrystal formation, we attempted to prepare CBZ-NIC cocrystal and CBZ-SAC cocrystal in a 1:1 volume ratio ethanol-water solvent mixture and polyvinyl pyrrolidone (PVP) solution. Cocrystal screening products were characterized by FTIR, DSC, and PXRD techniques. Characterization results show that in an ethanol-water solvent mixture, pure CBZ-NIC cocrystal can be prepared, while CBZ-SAC cocrystal cannot be formed. The addition of PVP can inhibit CBZ-NIC cocrystal formation and facilitate CBZ-SAC cocrystal formation, which could be the result of integration of the solubility enhancement ability of PVP and the intermolecular interaction between PVP and cocrystal components. Polymers are complicated macromolecules; their properties vary with the monomer species, polarity, molecular weight, and distribution, etc. The properties of pharmaceutical cocrystals also vary a lot with different APIs or CCFs. Thus, the conclusion regarding the influences of polymers on pharmaceutical cocrystals formation cannot be perfectly summarized based on limited experiments. Further research is necessary to supplement and improve the conclusions.

## Figures and Tables

**Table 1:** Solubility of carbamazepine (CBZ), Nicotinamide (NIC), and saccharin (SAC) in different solvents (mol fraction); PVP (polyvinyl pyrrolidone).

API/CCF	Water	Ethanol	Ethanol-Water	Ethanol-Water + PVP
CBZ	0.00001	0.00500	0.00011	0.00014
NIC	0.09242	0.17656	0.01961	0.02963
SAC	0.00032	0.00931	0.00127	0.00141
